# Increased Placental Phospholipid Levels in Pre-Eclamptic Pregnancies

**DOI:** 10.3390/ijms14023487

**Published:** 2013-02-06

**Authors:** Xiao Huang, Arjun Jain, Marc Baumann, Meike Körner, Daniel Surbek, Peter Bütikofer, Christiane Albrecht

**Affiliations:** 1 Institute of Biochemistry and Molecular Medicine, University of Bern, Bern 3012, Switzerland; E-Mails: xiao.huang@ibmm.unibe.ch (X.H.); arjun.jain@ibmm.unibe.ch (A.J.); peter.buetikofer@ibmm.unibe.ch (P.B.); 2 Swiss National Center of Competence in Research, NCCR TransCure, University of Bern, Bern 3012, Switzerland; E-Mails: marc.baumann@insel.ch (M.B.); daniel.surbek@insel.ch (D.S.); 3 Department of Obstetrics and Gynecology, University Hospital, University of Bern, Bern 3010, Switzerland; 4 Institute of Pathology, University of Bern, Bern 3010, Switzerland; E-Mail: meike.koerner@pathology.unibe.ch

**Keywords:** phospholipids, pre-eclampsia, placenta

## Abstract

Physiological pregnancy is associated with an increase in lipids from the first to the third trimester. This is a highly regulated response to satisfy energy and membrane demands of the developing fetus. Pregnancy disorders, such as pre-eclampsia, are associated with a dysregulation of lipid metabolism manifesting in increased maternal plasma lipid levels. In fetal placental tissue, only scarce information on the lipid profile is available, and data for gestational diseases are lacking. In the present study, we investigated the placental lipid content in control *versus* pre-eclamptic samples, with the focus on tissue phospholipid levels and composition. We found an increase in total phospholipid content as well as changes in individual phospholipid classes in pre-eclamptic placental tissues compared to controls. These alterations could be a source of placental pathological changes in pre-eclampsia, such as lipid peroxide insult or dysregulation of lipid transport across the syncytiotrophoblast.

## 1. Introduction

Physiologic pregnancy is associated with a broad spectrum of metabolic adaptations, including increased metabolism of lipids and lipoproteins. Previous studies have found that there is an increase in triglycerides, total cholesterol and low-density lipoprotein (LDL) levels during pregnancy [1–4]. Elevated lipid levels may serve as an energy store to fulfill maternal and fetal metabolic needs, while maternal hypertriglyceridemia, especially towards late gestation, has an important role as a source of triglycerides for milk formation just before parturition [3].

On the other hand, elevated maternal triglyceride levels during early pregnancy have been associated with pregnancy complications, such as pre-eclampsia [4,5]. Pre-eclampsia is a human pregnancy-specific disorder that not only adversely affects maternal vascular function, but also fetal intrauterine growth [6]. It is a major cause of maternal and perinatal morbidity and mortality [7]. A study by Vrijkotte *et al.* found that an increase in maternal blood triglyceride levels was linearly associated with an increased risk of pre-eclampsia [5]. It is suggested that the rise in circulating triglycerides may be due to an increase in hepatic lipase activity and a decrease in lipoprotein lipase activity. Hepatic lipase is responsible for an increased synthesis of triglycerides at the hepatic level, whereas a decreased activity of lipoprotein lipase is responsible for reduced catabolism at the adipose tissue level [1], resulting in a net increase in circulating triglycerides.

Other studies have found that in addition to elevated triglyceride levels, women who subsequently developed pre-eclampsia had significantly higher blood concentrations of total cholesterol and LDL-cholesterol, and increased LDL/HDL ratios compared to control subjects [2,4,8,9]. Although maternal cholesterol is an important source of cholesterol for the fetus during early gestation, its importance becomes minimal during late pregnancy, due to the high capacity of fetal tissues to synthesize cholesterol [10].

A previous study demonstrated that placental fatty acid composition is determined by the fatty acid content in the maternal liver and the maternal plasma [11]. Therefore, it is likely that higher concentrations of total cholesterol and LDL-cholesterol, and increased triglycerides and LDL/HDL ratios in pre-eclampsia, are reflected in the placental lipid profile.

Changes in cholesterol to phospholipids (Ch:Pl) ratios in the placenta have important consequences for placental function and transport. A previous study showed that the Ch:Pl ratio of the syncytiotrophoblast basal plasma membrane decreases during pregnancy [12]. Transplacental transport of nutrients from mother to fetus is mediated across the syncytiotrophoblast basal plasma membrane [13]. Transport activities together with other membrane-associated properties, such as permeability, activities of enzymes and receptors, and stability are greatly influenced and modulated by the physical state of the membrane lipid bilayer and by protein-lipid interactions [12,14]. The microviscosity (η-) of lipid regions, a key factor influencing membrane fluidity [15], is determined by the molar ratio of Ch:Pl. Under physiological conditions and with naturally occurring phospholipids, an increase in Ch:Pl ratio results in an increase in η-, which in turn decreases membrane fluidity [14,16,17]. By virtue of its structure, cholesterol can effectively modulate the physical state of the phospholipid bilayer. It lowers membrane fluidity by decreasing fatty acyl chain mobility [12]. Therefore, increased cholesterol levels in pre-eclampsia could reduce membrane fluidity and hence disrupt transport across the placental trophoblast.

A study by Staff *et al.* [18] reported increased contents of phospholipids and cholesterol in the decidua basalis of women with pre-eclampsia. The decidua basalis is the uterine lining during pregnancy, which forms the maternal part of the placenta. It was suggested that an elevated lipid content of the decidua basalis might be the source of lipids causing maternal endothelial dysfunction in pre-eclampsia [18]. These lipids may also represent a source of the acute atherosis evident in the maternal spiral arteries in pre-eclampsia. However, the study did not determine the lipid profile of the placenta proper, *i.e.*, the fetal placenta. The only available data on the phospholipid composition of the placenta are restricted to physiological pregnancies [19,20], with no comparison to pathological cases.

In the present study, we investigated the lipid content of the placenta proper, with a focus on phospholipid levels and composition, in control *versus* pre-eclamptic samples. The findings provide novel insights on dysregulation of lipid metabolism and/or transport in the placenta of pre-eclamptic cases, with further implications for placental pathophysiology of this disorder.

## 2. Results and Discussion

### 2.1. Model Verification and Validation

To assure reliable data collection, different methods and sample collection protocols for placental tissue samples were reviewed and validated by (i) testing the reproducibility of the lipid isolation and measurement procedures and (ii) comparing tissue samples collected from different locations within the placenta to exclude differential lipid distribution.

To determine the between-runs assay imprecision (CV) including the extraction process, lipids were extracted in duplicates from nine adjacent parts of the same (control) placental sample and analyzed for cholesterol, sphingomyelin (SPH), phosphatidylcholine (PC), phosphatidylserine (PS), phosphatidylinositol (PI) and phosphatidylethanolamine (PE) in separate experiments done on nine different days (in duplicates). Analysis of the combined data shows that the CVs for all parameters were <17% (Table 1). To exclude major differences in the method performance between different isolations, an independent internal control sample was included in all runs. If the variation of the internal control was >25%, the whole extraction and measurement procedure was repeated. Moreover, in all analyses placental samples of both healthy controls and pre-eclamptic patients were included.

To determine potential physiological differences in the lipid distribution within the placenta, samples were collected from the central (C), paracentral (medial, M) and peripheral (lateral, L) parts of six different (control) placentas and evaluated with respect to their cholesterol and phospholipid composition (Table 1). The results showed no significant difference (paired *t*-test, *p* > 0.05) in any of the parameters tested between C, M and L locations. For all subsequent lipid analyses, samples of the central localization of term and pre-eclamptic placentas were used.

### 2.2. Increased Lipid Content in Pre-Eclampsia

Previous research on lipids during pregnancy has focused mainly on maternal blood, and thus little data is available on the lipid profile in placental tissue. The main objective of this study was to characterize and quantify selected lipid groups, especially phospholipid classes, in control *versus* pre-eclamptic placentas. The demographics and clinical characteristics of the pre-eclamptic patients and healthy controls are listed in Table 2. Both groups were comparable with regard to baseline characteristics such as age, parity and BMI. Although preterm placentas would more closely match the gestational age, and, related to this, birth weight and placental weight of the pre-eclamptic samples, they are commonly associated with pathological processes [21,22]. In addition, previous studies have shown labor induces significant cellular stress within the placenta [23] and non-labored preterm healthy placentas delivered by elective caesarean section are very difficult to obtain. Therefore, healthy term placentas were selected as controls.

In this study, we found an increased lipid content in the placenta proper in pre-eclamptic patients compared to normotensive controls (Table 3). The mean cholesterol levels were increased in pre-eclampsia compared to controls, however, the increase was not statistically significant (Table 3). Total phospholipid levels, calculated as the sum of the five classes, were increased in pre-eclampsia compared to term control samples. As discussed above, previous studies have found an increase in triglycerides, total cholesterol and low-density lipoprotein (LDL) levels during pregnancy [1–4]. We had access to two pre-term controls, and found that the general lipid content was lower in pre-term placentas compared to term controls (data not shown), thus being representative of the above mentioned gestational age effect. Therefore, the finding that the pre-eclamptic group shows a significantly higher phospholipid level than normotensive term controls, despite the lower gestational age of the former, strongly suggests a dysregulation in lipid metabolism and/or transport in pre-eclampsia. It is tempting to speculate that this effect would be even enhanced when compared to age-matched pre-term controls.

**Figure 1:**
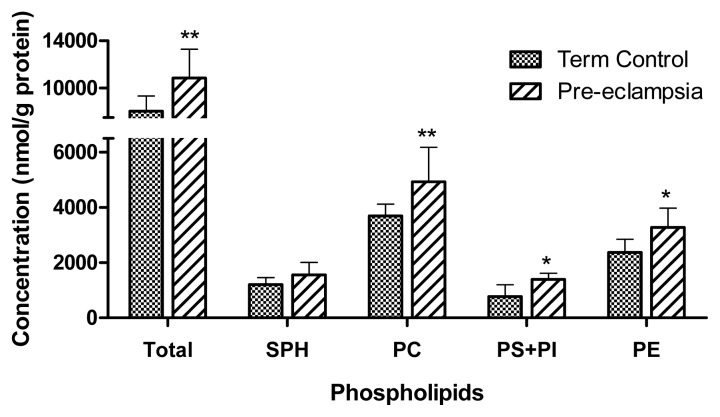
Total phospholipid content and distribution of individual phospholipid classes in control and pre-eclamptic placentas. Lipids were extracted from control (*n* = 9) and pre-eclamptic (*n* = 9) placentas and subsequently analyzed for individual phospholipid classes by thin layer chromatography as described in the Experimental Section. Total phospholipid content was determined as the sum of the individual phospholipid classes. Statistical analysis was performed using Mann-Whitney test. All values are expressed as mean ± SD. The level of statistical significance was set at *p < 0.05;**p < 0.01. SPH, sphingomyelin; PC, phosphatidylcholine; PS, phosphatidylserine; PI, phosphatidylinositol; PE, phosphatidylethanolamine.

Phospholipids comprise different glycerophospholipid and sphingophospholipid classes, including SPH, PC, PS, PI and PE [26] and their composition varies between tissues and membranes [27]. In our study, we determined the following phospholipid composition in placenta: SPH, 14%–15%; PC, 45%; PS + PI, 10%–11%; PE, 29%–30%. These values are consistent with previous studies on the phospholipid composition of the human placenta obtained from autopsies [20]. Table 3 outlines the individual phospholipid compositions of placental tissues obtained from term control subjects and from pre-eclamptic pathological patients. The results show that the levels of PC (*p* = 0.0085), PS + PI (*p* = 0.0120), and PE (*p* = 0.0264) were significantly increased in pre-eclamptic placenta compared to control tissue (Table 3, Figure 1). As shown in Figure 1, the concentrations of total phospholipids and phospholipid classes were increased by approx. 30%, and the content of PS + PI was even enhanced by approx. 80% in pre-eclamptic compared with control samples.

### 2.3. Implications of Increased Placental Lipid Content in Pre-Eclampsia

Although the etiology and pathophysiology of pre-eclampsia is still not fully understood, key pathophysiological characteristics include maternal vasospasm, placental ischemia, endothelial dysfunction, activation of blood coagulation, and an increased inflammatory response [28–30]. The initial event in the development of pre-eclampsia has been associated with deficient conversion of the uterine spiral arteries, which gives rise to an ischemia-reperfusion type insult. This is thought to be the underlying source of oxidative stress in pre-eclamptic pregnancies [31]. Oxidative stress induces the release of a complex mix of factors, including pro-inflammatory cytokines, apoptotic debris and angiogenic regulators [23,32,33], which are all implicated in the pathophysiology of pre-eclampsia. Lipid peroxides are also produced under oxidative stress conditions, when free radicals attack polyunsaturated fatty acids and cholesterol in membranes and lipoproteins. They are highly reactive compounds that can cause cellular dysfunction via different mechanisms, including direct interaction with cell membranes and an activation of redox-sensitive genes [34]. A previous study showed that phospholipid, total cholesterol and lipid peroxide levels were all elevated in the decidua basalis of pre-eclamptic samples compared to normal pregnancy samples [18]. It was suggested that the elevated lipid content of the decidua basalis could be directly responsible for maternal endothelial dysfunction in pre-eclampsia. These compounds could also be a source of the acute atherotic lesions evident in the maternal spiral arteries in pre-eclampsia. Our results show for the first time that phospholipid levels of the placenta proper are also increased in pre-eclamptic tissue compared to normotensive controls, and this could also be a source of oxidative damage within the placental parenchyma and associated placental pathophysiology in pre-eclampsia.

In our study, lipids were increased in pre-eclamptic tissue compared to term control samples. This included cholesterol, triglyceride, and phospholipids. Though the increase of cholesterol and triglycerides (data not shown) was not statistically significant in whole placental tissue, these results are reflective and in agreement with previous studies showing that an increased lipid profile of maternal blood during early pregnancy is positively correlated to an increased risk of pre-eclampsia.

Although the bulk of fetal cholesterol is synthesized *de novo* in fetal organs, maternal cholesterol may also be transported across the placenta and represents an additional source for fetal cholesterol [35]. The study by Scholler *et al.* describes a role for the phospholipid transfer protein (PLTP) in reverse cholesterol transport in the placenta. A previous study also found that fatty acid streaks have been found in fetal aortas, and their number was increased by maternal hypercholesterolemia [36]. Therefore, the increased placental lipid content we found in the pre-eclamptic samples likely represents the increased maternal lipid profile found in pre-eclampsia by previous studies.

One of the hypotheses associated with the pathogenesis of pre-eclampsia is based on the presence of hypercoagulation in the placental circulation. Previous studies have also linked a hypercoagulable state (thrombophilia) to severe obstetric pathology, such as intrauterine fetal death (IUFD) [37]. The negatively charged phospholipids are thought to be crucial components of the prothrombinase complex in the coagulation cascade by providing a catalytic surface on which coagulation proceeds [38,39]. Based on this known role of phospholipids, Omatsu *et al.* have established a pre-eclampsia-like model with hypercoagulation induced by injecting PC/PS (80%/20%) microvesicles into pregnant mice [40]. Using this murine model, the pre-eclampsia-like symptoms, such as increased blood pressure, decreased blood platelets and proteinuria, were considerably improved by anticoagulant agents (e.g., annexin V) [41]. Together these data indicate that activation of blood coagulation caused by phospholipid increase may contribute to the development of pre-eclampsia. Consistent with this model, our results demonstrate that phospholipid levels are significantly increased in the pre-eclamptic placenta. However, the relative composition of the four major phospholipid classes remained unchanged, indicating that the increase in phospholipid content is not caused by up-regulation of synthesis of a single phospholipid class but due to a general increase in phospholipid synthesis, or accumulation.

As the placenta is responsible for nutrient and waste exchange between mother and fetus, placental transport and metabolism of lipids are critical events for fetal development and survival. Biochemical abnormalities in lipid metabolism may account for the disrupted transport across the syncytiotrophoblast in pre-eclampsia. Indeed, previous studies have found impaired transport systems across the placenta in pathological pregnancies [42,43]. As described above, cholesterol can effectively modulate the physical state of the phospholipid bilayer by decreasing fatty acyl chain mobility and thereby lowering membrane fluidity [11]. It can be hypothesized that the trend towards an increased cholesterol content found in this study in pre-eclamptic placental tissue is reflective of an increase in cholesterol in the syncytiotrophoblast basal cell membrane, which in turn may affect membrane fluidity and compromise transport across the placenta. A potential increase in the cholesterol content of the syncytiotrophoblast basal cell membrane could be masked when whole placental tissue is examined and may be one reason why we did not observe a significant increase in the Ch:Pl ratio in pre-eclamptic *versus* control placentas.

Cholesterol and phospholipid efflux from various tissues is mediated via ATP-binding cassette (ABC) transporters, in particular ABCA1 [44–48]. ABCA1 is highly expressed in the placenta [46–48] and was suggested to play a role in cholesterol transfer from the maternal to the fetal circulation [49]. Moreover, previous studies from our laboratories demonstrated a reduced ABCA1 protein expression in the syncytiotrophoblast of pre-eclamptic placental tissues by immunohistochemistry [50], and decreased placental mRNA expression in pre-eclampsia as compared to controls (unpublished data). Therefore the increased phospholipid (and cholesterol) content in pre-eclamptic samples could also be reflective of impaired ABCA1 function resulting in compromised cholesterol and phospholipid efflux. Hence defective cellular efflux systems could also represent a potential source of cholesterol and phospholipid accumulation in the pre-eclamptic placenta observed in this study.

## 3. Experimental Section

### 3.1. Placental Sample Collection

The study was approved by the cantonal ethical committee, Bern, Switzerland. Samples of placental tissues from controls (*n* = 9) and pre-eclamptic patients (*n* = 9) were collected after caesarean section at the Department of Obstetrics and Gynecology, University Hospital Bern, Switzerland. Informed consent was obtained from all pregnant women. The clinical characteristics of patients and controls are described in Table 2. Pre-eclampsia was defined (according to the International Society for the Study of Hypertension in Pregnancy) as gestational hypertension of at least 140/90 mmHg on two separate occasions ≥4 h apart, accompanied by significant proteinuria of at least 300 mg in a 24 h collection of urine arising de novo after the 20th week of gestation [24].

Tissues were collected from the villous tree within one hour after delivery. To minimize blood contamination, each piece of tissue was intensively washed in DPBS (Gibco, Paisley, UK). Tissue samples were then snap-frozen and stored at −;80 °C.

### 3.2. Lipid Isolation

Tissue samples (500–600 mg of material) were homogenized in 0.9% NaCl and centrifuged at 4 °C for 60 min at 1600× *g*. The pellet was weighed and extracted using a two-phase extraction protocol according to Bligh and Dyer [51]. The organic phase was split in half for (i) cholesterol determination and (ii) phospholipid analysis. The aqueous phase of the extract was re-extracted once and its organic phase analyzed to determine residual amounts of lipids. The results showed that this fraction contained less than 2% of total lipid. All results were related to the protein concentration of the pellet as determined with a BCA Protein Assay Reagent kit (Pierce, Rockford, IL, USA).

### 3.3. Cholesterol Determination

The extracted lipids were dissolved in phosphate buffered saline (PBS) containing 2% Triton X-100. Total cholesterol concentrations were measured using an enzymatic kit purchased from Roche (Cat. No. 11491458) according to the manufacturer’s instructions.

### 3.4. Analysis of Phospholipid Classes by Thin Layer Chromatography

To determine the phospholipid composition, lipid extracts were separated by one-dimensional thin-layer chromatography using chloroform:methanol:acetic acid:NaCl 0.9% (2:1:0.3:0.1) as solvent system. Phospholipid standards were applied on each plate to identify individual classes. Lipids were stained by iodine vapor, spots were scraped into glass tubes, and lipid phosphorus was determined as described before [52].

### 3.5. Statistical Analyses

Differences regarding the clinical characteristics between patients and controls were tested using the unpaired *t*-test. Differences in the lipid distribution within the placenta were assessed using the paired *t*-test. Differences in the placental content of cholesterol, total and individual classes of phospholipids, and Ch:Pl ratio between control and pre-eclamptic placentas were performed using the Mann Whitney test. All statistical analyses were performed using the Graphpad^®^ software package. In all analyses, a *p* value <0.05 was considered statistically significant.

## 4. Conclusions

In this study, we investigated the lipid content of the placenta in control *versus* pre-eclamptic samples. We found a significant increase in total and individual phospholipid classes in pre-eclamptic placental tissue compared to control samples. This shows for the first time that placental (phospho)lipid content is generally reflective of the elevated lipid profile previously found in the maternal circulation. The increased placental lipid profile in pre-eclampsia is also indicative of placental pathology of this pregnancy disorder, which includes oxidative stress-induced lipid peroxide insult and dysregulation of transport across the placental syncytiotrophoblast. Further functional and mechanistic studies are needed to give insights whether the increase in phospholipids is causally related with the development of pre-eclampsia, and/or is a consequence and downstream effect of altered phospholipid transporters activity.

## Figures and Tables

**Table 1:** Method validation.

	Cholesterol (μmol/g)	Phospholipids (nmol/g)	Ch:Pl ratio	SPH (nmol/g)	PC (nmol/g)	PS + PI (nmol/g)	PE (nmol/g)
*n* = 9 a							
Mean ± SD	4.45 ± 0.75	11148 ± 1403	0.40 ± 0.06	1478 ± 246	5133 ± 770	1300 ± 208	3381 ± 382
CV%	16.77	12.59	15.23	16.66	14.99	16.02	11.30

***n*= 6**^b^							

Mean ± SD (C)	4.43 ± 0.53	8514 ± 1767	0.54 ± 0.14	1355 ± 293	3613.843 ± 606	1339 ± 503	2342 ± 440
Mean ± SD (M)	4.60 ± 0.77	8331 ± 1995	0.57 ± 0.11	1338 ± 181	3918 ± 961	764 ± 388	2377 ± 457
Mean ± SD (L)	5.31 ± 1.89	7661 ± 2450	0.70 ± 0.17	1155 ± 434	3519 ± 1124	1213 ± 154	2156 ± 741

a Nine adjacent parts of the same (control) placental sample were extracted (in duplicate) and analyzed (in duplicate) for cholesterol, sphingomyelin (SPH), phosphatidylcholine (PC), phosphatidylserine (PS), phosphatidylinositol (PI) and phosphatidylethanolamine (PE) on different days. Details are described in the Experimental Section. CV: coefficient of variation. Phospholipids represent the calculated sum of the individual phospholipid classes (SPH + PC + PS + PI + PE).

b Central (C), paracentral (medial; M) and peripheral (lateral, L) parts of 6 different (control) placentas were extracted and analyzed for the parameters described under^a^. No statistical differences between the locations were found (paired *t*-test; *p* > 0.05). All results are related to the protein concentration of the pellet obtained after initial centrifugation (details see Experimental Section).

**Table 2:** Demographics and clinical characteristics of controls and patients prior to delivery (mean ± SD).

	Controls (*n* = 9)	Pre-eclampsia 1 (*n* = 9)	*p*-value
Baseline characteristics			

Age, y	31.81 ± 4.98	32.21 ± 4.94	0.87, NS
BMI, kg/m^2^	2.37 ± 0.43	2.35 ± 0.24	0.89, NS
Gravidity	2.71 ± 0.95	1.78 ± 0.97	0.07, NS
Parity	1.14 ± 1.07	0.33 ± 1.00	0.14, NS
GA, weeks	38.80 ± 1.12	28.57 ± 3.27	<0.0001
Birth weight, g	3400 ± 359	949 ± 470	<0.0001
Placental weight, g	617 ± 209	273 ± 146	<0.005
Smoking	13% (1/8)	0% (0/9)	
Caucasian	100% (8/8)	78% (7/9)	

**Clinical Data**			

Systolic BP, mmHg	112 ± 10	178 ± 22	<0.0001
Diaystolic BP, mmHg	62 ± 5	99 ± 12	<0.0001
Proteinuria, g/24 h	<0.3 2	1.66 ± 1.95	<0.0001

1 Pre-eclampsia was defined (according to the International Society for the Study of Hypertension in Pregnancy) as gestational hypertension of at least 140/90 mmHg on two separate occasions ≥4 h apart, accompanied by significant proteinuria of at least 300 mg in a 24 h collection of urine arising *de novo* after the 20th week of gestation and resolving completely by the 6th week postpartum [24]. This group (seven Caucasian, one Hispanic, one African patient) contains seven cases with intrauterine growth restriction (IUGR) defined by birth weight <10th percentile [25].

2 Non-significant proteinuria as determined by 24 h-urine analysis or <1+ on dipstick. *BMI*, (preconceptional) body mass index*; GA* gestational age (at delivery); *BP*, blood pressure; *NS*, not significant. Statistical analysis was performed using an unpaired Student’s *t*-test.

**Table 3:** Placental lipid composition.

	Term control (*n* = 9)	Pre-eclampsia (*n* = 9)
Cholesterol (μmol/g)	4.16 ± 0.83	4.41 ± 0.69
Phospholipids (nmol/g)	8057.01 ± 1266.65	10850.85 ± 2431.27 **
Ch:Pl ratio	0.39 ± 0.16	0.40 ± 0.15
SPH (nmol/g)	1212.80 ± 243.62	1558.91 ± 455.06
PC (nmol/g)	3693.99 ± 428.16	4932.25 ± 1249.12 **
PS+PI (nmol/g)	774.50 ± 428.31	1389.92 ± 232.07 *
PE (nmol/g)	2375.71 ± 470.87	3275.87 ± 700.34 *
SPH:PC:(PS+PI):PE	15:46:10:29	14:45:11:30

Placental samples from the central location were extracted (in duplicate) and analyzed (in duplicate) for cholesterol, sphingomyelin (SPH), phosphatidylcholine (PC), phosphatidylserine (PS), phosphatidylinositol (PI) and phosphatidylethanolamine (PE) as described in the Experimental Section. Cholesterol and phospholipid results are related to the protein concentration of the pellet obtained after initial centrifugation (details see Experimental Section); all data are presented as mean ± SD. *Phospholipids* represent the calculated sum of the individual phospholipid classes (SPH + PC + PS + PI + PE). *Ch:Pl ratio*, cholesterol: phospholipids ratio. Statistical analysis was performed using Mann-Whitney test. The level of significance was set at * *p* < 0.05; ** *p* < 0.01.
